# Novel *Streptococcus suis* Sequence Type 834 among Humans, Madagascar

**DOI:** 10.3201/eid2402.171138

**Published:** 2018-02

**Authors:** Mihaja Raberahona, Saïda Rasoanandrasana, Vonintsoa Lalaina Rahajamanana, Felana Ranaivo-Rabetokotany, Volatiana Andriananja, Fetra Angelot Rakotomalala, Mamy Jean de Dieu Randria, Luc Rakotovao, Corinne Marois-Créhan, Véronique Tocqueville, Fabrice Touzain, Mala Rakoto-Andrianarivelo

**Affiliations:** Hôpital Universitaire Joseph Raseta Befelatanana, Antananarivo, Madagascar (M. Raberahona, S. Rasoanandrasana, F. Ranaivo-Rabetokotany, V. Andriananja, M.J.D. Randria, L. Rakotovao);; Hôpital Universitaire Mère-Enfants Tsaralalàna, Antananarivo (V.L. Rahajamanana);; Centre d’Infectiologie Charles Mérieux, Antananarivo (F.A. Rakotomalala, M. Rakoto-Andrianarivelo);; Agence Nationale de Sécurité Sanitaire de l’Alimentation, de l’Environnement et du Travail, Ploufragan, France (C. Marois-Créhan, V. Tocqueville, F. Touzain)***;***; Université Européenne Bretagne-Loire, Rennes, France (C. Marois-Créhan, V. Tocqueville, F. Touzain)

**Keywords:** *Streptococcus* *suis*, *Streptococcus pneumoniae*, human infection, swine, pig, farm, pork meat, ST834, whole-genome sequencing, bacterial meningitis, Madagascar, Togo, Africa, bacteria, meningitis/encephalitis

## Abstract

Two cases of meningitis caused by *Streptococcus suis* occurred in Madagascar, 1 in 2015 and 1 in 2016. We report the characterization of the novel sequence type, 834, which carried the *mrp*+/*sly*+/*epf*+ virulence marker and a mutation G→T at position 174, leading to a substitution mutS1 to mutS284.

*Streptococcus* *suis* is a common pathogen among pigs that can be transmitted to humans, in whom it causes invasive infection. In recent years, cases among humans have been reported worldwide, and a large outbreak occurred in China in 2005 (*1*). In some areas, *S*. *suis* appeared as the most common etiology of adult meningitis (*2*). Data from Africa were scarce until the recent report of 15 cases in Togo (*3*). We report 2 cases of *S*. *suis* meningitis in Antananarivo, Madagascar.

In March 2015, a 24-year-old man (patient 1) was admitted to the Infectious Diseases Unit of Befelatanana Hospital (Antananarivo) seeking treatment for fever, headache, and unilateral sixth nerve palsy. One year later, in March 2016, a 60-year-old woman (patient 2) was admitted to the same unit with meningitis and sudden hearing loss. Patient 1 worked in a slaughterhouse and patient 2 as a cook; both were frequently exposed to pork meat. Both patients were febrile (temperature >39.9°C) and confused and had a score of <13 out of 15 on the Glasgow Coma Scale. Laboratory results for lumbar puncture showed turbid cerebrospinal fluid (CSF) with increased cell numbers (446 cells/µL, 56% neutrophils for patient 1; 1,180 cells/µL, 86% neutrophils for patient 2 [reference range <10/µL]); high protein levels (1.4 g/L for patient 1, 2.34 g/L for patient 2 [reference range 0.15–0.45 g/L]); and low glucose levels (0.28 mmol/L for patient 1; 0.73 mmol/L for patient 2 [reference range 2.7–4.2 mmol/L]). CSF Gram stain results showed gram-positive diplococci resembling *Streptococcus pneumoniae*. CSF samples inoculated onto chocolate agar showed microbial growth that was further found to contain optochin-resistant α-hemolytic streptococci. In-house real-time PCR on CSF and colonies, targeting the specific gene for *S. pneumonia* were negative. The colonies were finally identified as *S. suis* by using ApiStrep20 (bioMérieux, Marcy l’Étoile, France) and matrix-assisted laser desorption/ionization time-of-flight mass spectrometry with a log (score) value of spectra >2.3 (cutoff ≥2.0). Both patients achieved overall recovery after a complete course of intravenous ampicillin (patient 1) or ceftriaxone (patient 2), but a mild hearing deficit acquired during the infection remained for both patients.

We retrieved only strain Ss1223, isolated from patient 2, for further investigation. By using slide agglutination with type-specific hyperimmune serum and specific multiplex PCR (*4*), we identified the Ss1223 strain as *S. suis* serotype 2. We performed whole-genome sequencing by using an Ion Torrent Proton sequencer (Thermo Fischer Scientific, Waltham, MA, USA). After cleaning data with Trimmomatic 0.36 (*5*), we align reads of S735 *S. suis* sequence type ST1 European reference strain (GenBank accession no. NC_018526.1) by using the maximal exact matches algorithm of Burrows-Wheeler Aligner software version 0.7.15-r1140 (https://omictools.com/burrows-wheeler-aligner-tool), downsampled to fit an estimated coverage depth of 80× before assembly. SPAdes 3.8.1 (*6*) assembly was deposited in GenBank (accession no. NHOL00000000). By applying the previously described pipeline (*7*), we confirmed the Ss1223 strain as serotype 2 and a novel sequence type (ST), 834, bearing the commonly occurring virulence markers of *S. suis* among persons who handle pork meat, *mrp*+/*sly*+/*epf*+. The *epf* gene contains a single-nucleotide polymorphism and 2 insertions/deletions. Moreover, ST834 carries the mutation G→T at position 174, leading to a substitution *mutS*1 to *mutS*284 (http://pubmlst.org/ssuis/), an element involved in the mismatch repair mechanism that contributes to maintaining the overall fidelity of DNA replication. These findings indicate that ST834 is closely related to ST1, which is among the most prevalent and virulent *S. suis* clones worldwide.

We report emergence of human *S. suis* infection in Madagascar and describe the epidemiology of *S. suis* in Africa, in addition to research conducted in Togo recently (*3**,**8*). Both case-patients were at risk for infection because of their professional occupations (*9*). The diagnosis would have been missed if there had been no in-depth discussion between clinicians and biologists and if thorough laboratory investigation by using conventional biochemical methods has not been done. Because *S. suis* infection is not commonly known as an etiology of bacterial meningitis in Madagascar and may be misidentified as other streptococcal infections if results are based on culture results alone (*10*), we also used matrix-assisted laser desorption/ionization time-of-flight mass spectrometry.

In Madagascar, pig farming is concentrated in the central highlands and depends on small holders. Pig slaughtering is not always done in abattoirs and does not necessarily follow good rearing practices. Thus, susceptible persons may be exposed to infectious organisms that could cause outbreaks.

The emergence of a novel *S. suis* ST carrying virulence markers raises questions about the zoonotic potential of this pig pathogen, suggesting that further study on *S. suis* circulation in pigs will be useful for an understanding of its association with these and other human cases. Our findings also provide evidence that whole-genome sequencing is an indispensable tool for studying the genetic diversity of *S. suis*, detecting the emergence of novel sequence types and characterizing virulence factors. In conclusion, our study highlights the need to increase awareness of *S. suis* infections among clinicians and laboratory staff and to implement a surveillance system for both pigs and humans that includes the emerging ST834 strain.
